# The characteristics of choriocapillaris flow void in the unilateral polypoidal choroidal vasculopathy fellow eyes

**DOI:** 10.1038/s41598-021-02377-x

**Published:** 2021-11-29

**Authors:** Huajui Wu, Yukinori Sugano, Kanako Itagaki, Akihito Kasai, Hiroaki Shintake, Tetsuju Sekiryu

**Affiliations:** Department of Ophthalmology, Fukushima Medical University, 1 Hikariga-oka, Fukushima, Fukushima Japan

**Keywords:** Retinal diseases, Predictive markers, Eye manifestations

## Abstract

To evaluate the morphological characteristics of flow void (FV) in the fellow eyes of the unilateral polypoidal choroidal vasculopathy (PCV). Fifty PCV fellow eyes (PCVF) and 31 age-matched normal ocular circulation controls were recruited in this retrospective study. The number of FV was analyzed according to the size in a centered 5 × 5 mm swept source optical coherence tomography angiography scans. We used indocyanine green angiography images to determine whether choroidal vascular hyperpermeability (CVH) has occurred. For the PCVF, the prevalence rate of CVH was 70% (35 of 50) The number of FVs was significantly lower in 400–25,000 μm^2^ (P = 0.005), 400–500 μm^2^ (P = 0.001), 525–625 μm^2^ (P = 0.001) and 650–750 μm^2^ (P = 0.018). compared to the controls. And showed no difference in size from 775 to 1125 μm^2^ between the two groups. The area under the receiver operating characteristic curve of PCVF with CVH and controls was 0.94 (95% CI 0.88–1.00) (P < 0.001). We found that the number of small FVs was significantly lower in the PCV fellow eyes than that in the eyes with control group.

## Introduction

Polypoidal choroidal vasculopathy (PCV) is a distinctive clinical entity that may cause choroidal neovascularization (CNV) in elderly people as proposed by Yannuzzi in 1990. PCV is usually diagnosed by indocyanine green angiography (ICGA) examination, which detects polypoidal lesions or by indirect ophthalmoscopy, which identifies orange nodules structures^1,2^. The genetic predisposition may affect the pathophysiology of the PCV^3^. The fellow eyes of the PCV may be exposed to the background factors. And the PCV unaffected fellow eyes sometimes show thick choroid features, despite unilateral cases. These facts suggest that pathognomonic changes may already begin in the fellow eyes of PCV. Analysis of PCV fellow eyes may provide a clue to elucidate the pathogenesis of PCV.

The choroidal vasculature of PCV shows distinctive changes such as enlargement of vessels in the Harller’s layer or attenuation of small vessels in the Sattler’s layer. The changes of the choriocapillaris (CC) in PCV would be expected. However, it has been reported^4^ that there was no difference in vascular parameters of the choroidal vasculature, including the CC between the pachychoroid eyes without disease and normal eyes in a study using optical coherence tomography angiography (OCTA). Other studies^5^ have found that the CC flow deficit of PCV fellow eyes decreased, but did not reach statistical difference compared to the age-matched control group. Since CC cannot be delineated directly with the current OCTA devices, careful consideration is required to reach a conclusion.

Swept-source optical coherence tomography angiography (SS-OCTA) is often used to evaluate CC 2D images, due to its high scanning speed for evaluation. Although it has a wide depth range, the lateral resolution is generally lower than the spectral-domain OCTA. For example, the lateral resolution of Plex Elite 9000 SS-OCTA (Carl Zeiss Meditec Inc, Dublin, California, USA), which was used in this research, was 20 μm^6^.

The diameter of normal CC (16–20 μm) is smaller than the instrument resolution^7–9^. We could not see the CC structures directly on the SS-OCTA image. We used CC flow void (FV) as a substitution for the CC structure, which was defined as the part of the CC slab without blood flow signals that diameter was bigger than the resolution of the SS-OCTA instrument.

Blood flow congestion in the choroid was postulated in PCV. We assumed that two possible changes were caused by congestion. One was that the enlargement of the capillary diameter due to congestion resulting FV decrease. The other was that ischemia due to congestion causing capillary dropout and FV increased. We speculated that the FV decrease that is due to congestion may appear in the small size of FV in early stage. Then it may increase in size later. To clarify this issue, we need to analyze FV sorted by size. To the best of our knowledge, an FV analysis by size in the eyes of subjects with PCV and fellow eyes has not yet been reported.

In the current study, we measured the number of FVs in groups stratified by the FV sizes to clarify the changes of FV in the fellow eyes of patients with unilateral PCV.

## Methods

### Patients

This was a retrospective comparative study that was conducted in accordance with the Declaration of Helsinki and was approved by the Institutional Review Board of Fukushima Medical University (No. 2020-091), the Institutional Review Board of Fukushima Medical University approved the waiver for individual informed consent for this analysis.

We reviewed the medical records of 862 Japanese patients who were diagnosed as having unilateral PCV between June 2017 and August 2020 at Fukushima Medical University Hospital, Fukushima, Japan.

The inclusion criterion was a diagnosis of unilateral PCV evaluated by three ophthalmologists based on the presence of branching vascular networks, polypoidal lesions, and dilated aneurysmal lesions on ICGA (TRC 50DX, Topcon, Tokyo, Japan) according to the published criteria^1,2^. Thirty-one fellow eyes of patients diagnosed as having unilateral retinal vein occlusion (RVO) were selected as an age-matched control group. All RVO patients underwent fluorescein angiography (FA) for clinical evaluation, and were confirmed to have no abnormality. there was no evidence of retinal pathological change in any of the participants in either group.

The exclusion criteria for all eyes were as follows: (1) eyes with any combined retinal disease or intraocular disease, such as a macular hole, central serous chorioretinopathy, uveitis, or glaucoma; (2) patients with any systemic disease that affects CC circulation, such as hypertension, diabetes, gout, hypercholesterolemia, or neoplasm, or are allergic to the ICGA dye; (3) the refractive error is greater than − 6 diopters or the axial length is over 27 mm; (4) any observed drusen, retinal pigment epithelium (RPE) abnormality, or segmentation errors on the B-scan of the SS-OCT images.

In which 773 PCV patients met the exclusion criteria 1–3 and has been excluded. We then reviewed the SS-OCT images of the remaining 89 patients, 39 of whom met exclusion criterion 4, and were excluded.

**Figure 1:**
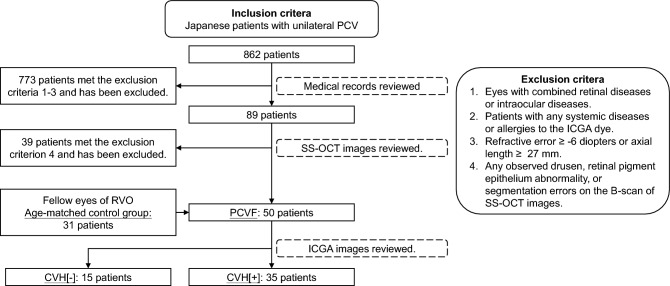
Flow chart of the patient extraction and grouping process. First, we included the medical records of all patients who had been diagnosed as having PCV in our hospital between June 2017 and August 2020. We checked the medical records for exclusion criterion 1–3. Then we checked the relevant images of each patient on the SS-OCT machine, finally including 50 PCV patients as the PCVF group. We selected the control group using the same steps. Finally, we checked the ICGA images of the PCVF group members and divided them into the CVH[+] and CVH[−] groups. *FV* choriocapillaris flow void, *PCVF* PCV fellow eyes. *Control* age-matched control group, *CVH[−]* PCV fellow eyes without choroidal vascular hyperpermeability, *CVH[+]* PCV fellow eyes with choroidal vascular hyperpermeability.

All patients who did not meet the exclusion criteria were enrolled. Fifty fellow eyes of 50 patients were categorized in the PCV fellow eyes (PCVF) group. According to the results of the choroidal vascular hyperpermeability (CVH) status, the PCVF group was further divided into two groups; PCV fellow eyes with CVH (CVH[+]) and PCV fellow eyes without CVH (CVH[−]). Thirty-one patients who were diagnosed as having unilateral retinal vein occlusion (RVO) were selected as controls (Fig. 1).

### Clinical examination

All enrolled patients underwent comprehensive ophthalmic examinations, including indirect ophthalmoscopy, slit-lamp biomicroscopy, color, and red-free fundus photography, SS-OCTA, and ICGA (PCVF)/or FA (control) at the first visit. RVO patients did not receive ICGA for ethical reasons.

**Figure 2:**
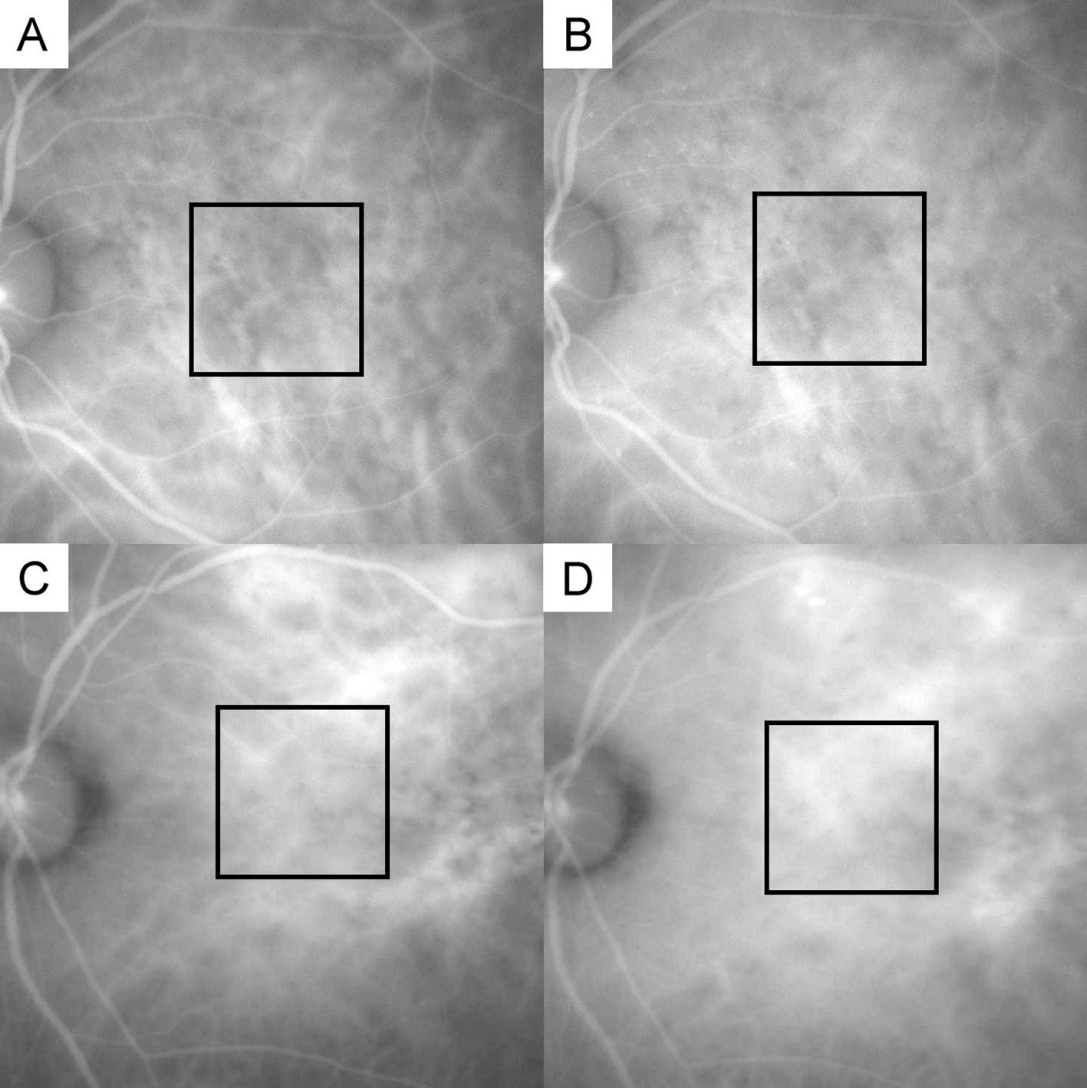
Representative images of choroidal vascular hyperpermeability status grading. The square represents a 5 × 5 mm^2^ area corresponding to the OCTA image. (**A**,**C**) ICGA images taken 5 min after the dye injection, as a benchmark for comparison. (**B**,**D**) ICGA images taken about 10 min after the dye injection, to evaluate the CVH status in the square. (**B**) Scored 1 (CVH[−]): compared with (**A**,**B**) has no choroidal vascular hyperpermeability. (**D**) Scored 2 (CVH[+]): compared with (**C**,**D**) has obvious and strong choroidal vascular hyperpermeability. CVH[−]: PCV fellow eyes without choroidal vascular hyperpermeability. CVH[+]: PCV fellow eyes with choroidal vascular hyperpermeability.

The CVH was defined as hyper fluorescence seen in the mid-phase of the ICGA images (10 min after the dye was injected), and the correspondent area of the OCTA images (Fig. 2).

The CVH was evaluated by two masked ophthalmologists. The 5 min images were as the reference and scored the ICGA images around 10 min. The scores were defined as follows. All participants of the control group were scored 0. A score of 1 was given when there was no distinguishable CVH in the area (CVH[−]); and a score of 2 was given when there was obvious and strong CVH in the area (CVH[+]). When the results were inconsistent, another experienced ophthalmologist made the judgment.

The subfoveal choroidal thickness (SFCT) was measured by masked ophthalmologists as the vertical distance between the RPE and the choroidoscleral border at the center of the fovea on the B-scan of the SS-OCT, using ImageJ software, version 1.52p (National Institutes of Health, Bethesda, Maryland, USA. https://imagej.nih.gov/ij/index.html^10^.

### Imaging processing

**Figure 3:**
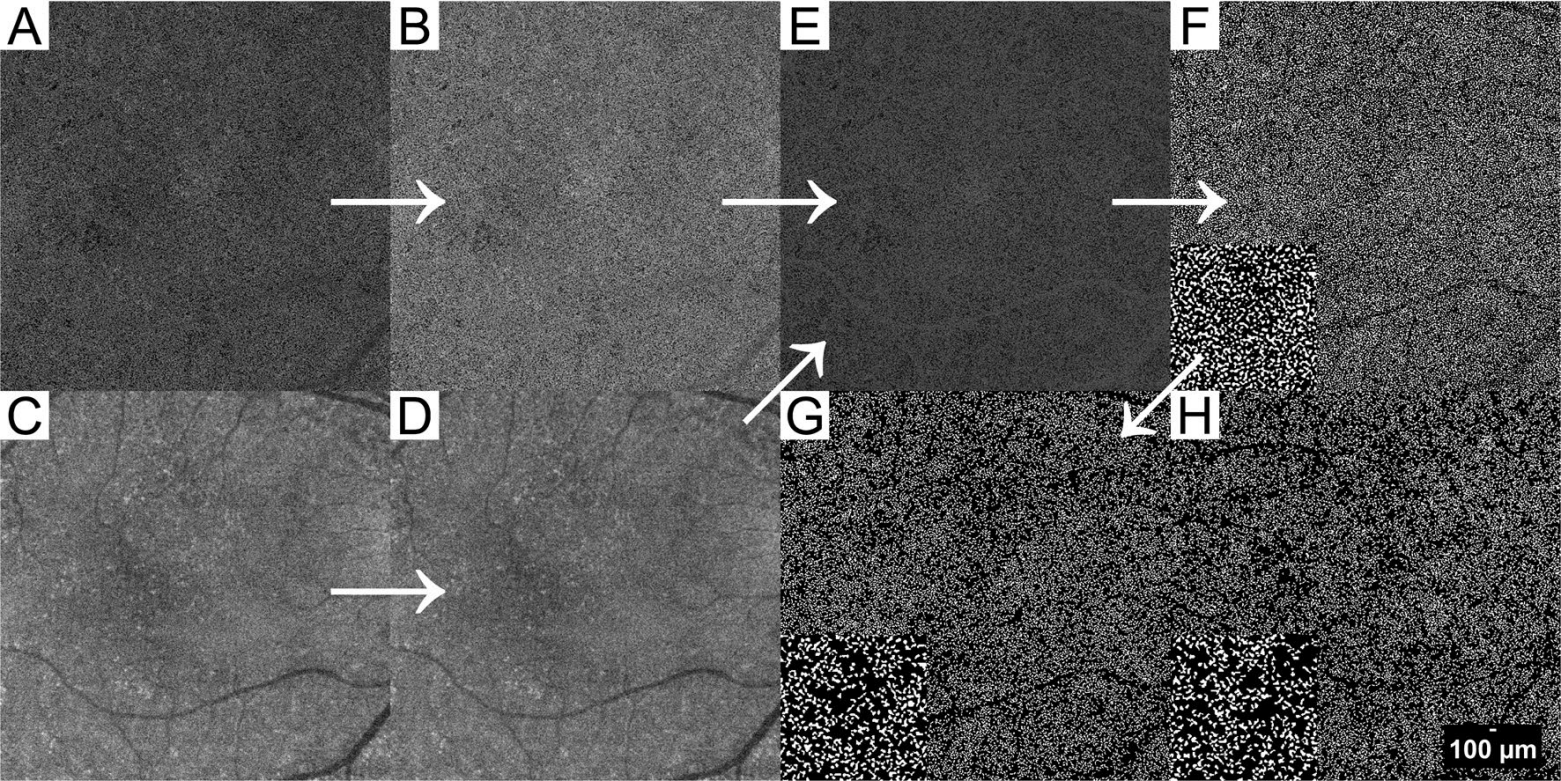
Demonstration of image processing protocol. A 64-year-old control eye. The white arrows demonstrate our image process protocol. (**A**,**C**) Original angiography image and a corresponded structural OCT image were exported from the SS-OCT machine. (**B**,**D**) Images after normalization and resizing. (**E**) After enhancement, the shadows of superficial vessels have been eliminated. (**F**) An FV image after binarization. White parts represent the FV and signal noise. Composition: 1 mm^2^ in the center of the image. The FV was incorrectly connected into clusters because of the signal noise. (**G**) Result after removing the noise. Composition: 1 mm^2^ in the center of the image. The FV was divided into reasonable sizes. (**H**) A 66 year old PCV fellow eye with CVH, showed sparse FV. Composition: 1 mm^2^ in the center of the image. *FV* choriocapillaris flow void, *CVH* choroidal vascular hyperpermeability.

To analyze the FV, we conducted the binarization of the CC slab and grouped the FV according to our previous research (Fig. 3)^11^.

We imported the CC slab bounded from 31 to 40 μm beneath the RPE reference^12^ and the correspondent en-face structural image to MATLAB R2019a. (The MathWorks Inc. Natick, Massachusetts, USA). A compensation algorithm was used to eliminate the influence of the shadowing effect on the angiography images^13^. We then imported the compensated images into ImageJ and processed the “Phansalkar local threshold” (radius = 5) for binarization^14^. After binarization, we processed "Analyze Particles" and “watershed irregular”^15^ to remove the signal noise.

Since the center of the macula of some subjects was displaced from the center of the image (− 210 to 455 μm), we performed manual correction to ensure that all images contained the same 5 × 5 mm^2^ area centered at the fovea. We applied "Analyze Particles" to summarize the FV in each interval.

### Statistical analysis

Statistical calculations were performed by IBM SPSS V.26 for Windows (IBM Co., Armonk, New York, USA). A P value of less than 0.05 was defined as statistically significant. The Kolmogorov–Smirnov test (K–S test) was performed to detect the normality of distribution. Because the K–S test of the FV number showed significance in most intervals, all variables are expressed as median and quartile deviation. The independent samples median test, adjusted by Bonferroni correction, was used to calculate the differences between the groups. Spearman's rank correlation coefficient was performed to evaluate the correlation between FV and CVH. The Youden index was used to determine the best cut-off point for the receiver operating characteristic (ROC) curve between the CVH[+] group and the controls.

### The size of FV analysis

We compared the number and distribution of FVs between the PCVF group and controls in the following intervals to confirm the difference between the fellow eyes of PCV and normal circulation eyes.

The primary outcome was the number of FVs in different intervals between PCVF group and controls.

The number of FVs was divided into the following seven groups.

FVall: the FV sizes ranged from 400 to 25,000 μm^2^ (16–1000 pixels). FV500: the FV sizes ranged from 400 to 500 μm^2^ (16–20 pixels). FV625: the FV sizes ranged from 525 to 625 μm^2^ (21–25 pixels). FV750: the FV sizes ranged from 650 to 750 μm^2^ (26–30 pixels). FV875: the FV sizes ranged from 775 to 875 μm^2^ (31–35 pixels). FV1000: the FV sizes ranged from 900 to 1000 μm^2^ (36–40 pixels). FV1125: the FV sizes ranged from 1025 to 1125 μm^2^ (41–45 pixels).

We calculated the total area of the FV sizes ranged from 400 to 25,000 μm^2^ for comparison (FVarea).

### The relationship between CVH and FV

To investigate the relationship between FV and CVH status, we performed Spearman's rank correlation analysis among the FV and CVH status in all intervals.

We then compared the number of FVs between CVH[+], CVH[−] and control groups in each interval to determine the influence of CVH.

### ROC analysis

We used binary logistic regression analysis for the CVH[+] and control groups depending on the results of the FV and CVH analyses.

FV smaller than 750 μm^2^ was grouped every 25 μm^2^ (1 pixel) and subjected to a binary logistic regression analysis after standardization.

We performed ROC analysis to determine a cut-off value for maximizing sensitivity and specificity to discriminate between the two groups.

## Results

### Baseline characteristics

The mean ages were 68.4 ± 7.8 (51–84 years) in the PCVF group and 67.7 ± 8.8 (55–84 years) in the control group. There were no differences observed between the two groups regarding gender (P = 0.105), age (P = 0.677), refractive error (P = 0.677), or subfoveal choroidal thickness (P = 0.234).

The prevalence rate of CVH was 70% in the PCVF group (35 of 50 participants.)

The demographic characteristics of the groups are shown in Table 1.

**Table 1:** Characteristics of participant groups (mean ± SD).

Groups	PCVF	Control	P value^†^
No. of eyes (No. of right eyes)	50 (27)	31 (14)	0.103
No. of male	40 (80.0%)	21 (67.7%)	0.105
Age (years)	68.4 ± 7.8	67.7 ± 8.8	0.671
Re (Diopter)	0.5 ± 1.8	0.3 ± 1.8	0.677
SFCT (μm)	241.3 ± 84.2	220.8 ± 55.9	0.234

*Re* refractive error, *SFCT* subfoveal choroidal thickness, *PCVF* PCV fellow eyes, *Control* age-matched control group.

^†^Student t-test.

### Validation of the normal distribution

The Kolmogorov–Smirnov test results between the two groups were as follow: age: P = 0.200; Refractive error: P = 0.200; SFCT: P = 0.200; FVall: P = 0.003; FV500: P < 0.001; FV625: P = 0.002; FV750: P = 0.023; FV875: P = 0.200; FV1000: P = 0.200; FV1125: P = 0.200; FVarea: P = 0.006.

### The number of FVs

**Figure 4:**
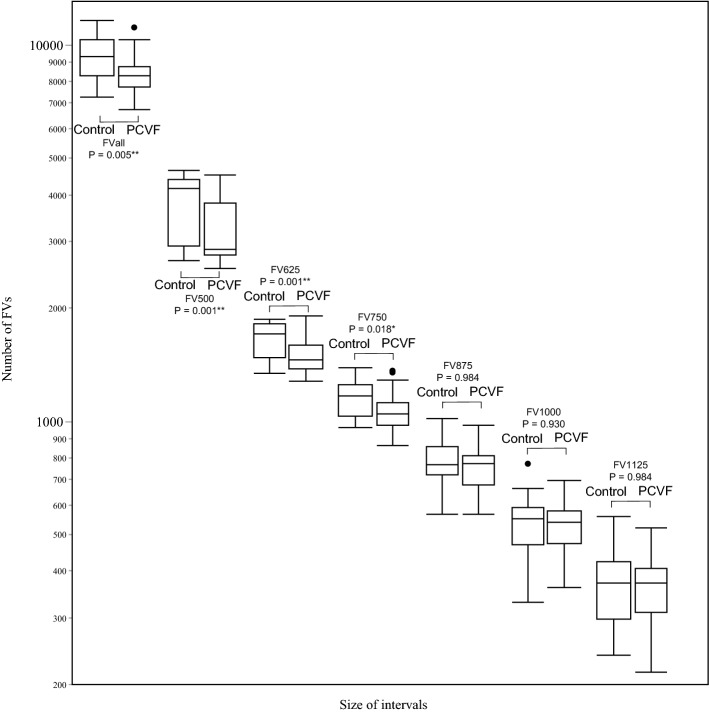
Comparison of FVs between PCV fellow eyes and controls. *P* P value of independent-samples median test. The number of FVs was significantly lower in the PCVF group compared to the controls in FVall, FV500, FV625, and FV750. There were no differences between the two groups in FV875, FV1000, and FV1125. *P < 0.05, **P < 0.01. *FV* choriocapillaris flow void, *Control* control group, *PCVF* PCV fellow eyes, *FVall* FV sizes from 400 to 25,000 μm^2^. FV500: FV sizes from 400 to 500 μm^2^. FV625: FV sizes from 525 to 625 μm^2^. FV750: FV sizes from 650 to 750 μm^2^. FV875: FV sizes from 775 to 875 μm^2^. FV1000: FV sizes from 900 to 1000 μm^2^. FV1125: FV sizes from 1025 to 1125 μm^2^.

The relationship between FV size and FV number is presented in Fig. 4 and Table 2.

**Table 2:** The number of the flow void in different sizes.

Intervals of FV sizes	Groups						P value
	Control (n = 31) (Median, QD)		PCVF (n = 50) (Median, QD)		95% CI		Median Test^†^
FVall (0–25,000 μm^2^)	9297.0, 2063.0		8260.0, 1040.0		6797.7–11,191.3		0.005**
FV500 (400–500 μm^2^)	4153.0, 1455.0		2857.5, 1037.0		2568.4–4630.5		0.001**
FV625 (525–625 μm^2^)	1704.0, 335.0		1461.0, 214.3		1299.5–1870.6		0.001**
FV750 (650–750 μm^2^)	1170.0, 224.0		1050.0, 139.8		896.9–1363.3		0.018*
FV875 (775–875 μm^2^)	768.0, 134.0		774.0, 133.3		569.0–975.6		0.984
FV1000 (900–1000 μm^2^)	551.0, 119.0		541.0, 103.3		361.2–696.4		0.930
FV1125 (1025–1125 μm^2^)	372.0, 126.0		373.0, 96.3		230.5–521.9		0.984
FVarea (μm^2^)	6.1,1.1		6.2, 1.2		4.6–8.5		0.586

*QD* quartile deviation, *Control* age-matched control group, *PCVF* PCV fellow eyes.

^†^Independent-samples median test. *P < 0.05, **P < 0.01.

There were no differences in FVarea between the two groups (P = 0.586).

In a comparison between the PCVF and control groups, the number of FVs in the PCVF group at all intervals was lower than that in the control group. There was statistical significance between the PCVF and the control group in the following intervals; FVall (P = 0.005), FV500 (P = 0.001), FV625 (P = 0.001) and FV750 (P = 0.018). No difference was observed between the two groups in FV875 (P = 0.984), FV1000 (P = 0.930) or FV1125 (P = 0.984).

### The analysis between FV and CVH

In a comparison among all participants, the number of FVs in the following intervals was significantly negative correlated with the CVH status: FVall (r = − 0.39, P < 0.001), FV500 (r = − 0.45, P < 0.001), FV625 (r = − 0.36, P = 0.001) and FV750 (r = − 0.30, P = 0.006) (Table 3).

**Table 3:** The correlation between choroid vascular hyperpermeability and flow void.

Intervals of FV sizes	r	P value^†^
FVall (0–25,000 μm^2^)	− 0.39	< 0.001**
FV500 (400–500 μm^2^)	− 0.45	< 0.001**
FV625 (525–625 μm^2^)	− 0.36	0.001**
FV750 (650–750 μm^2^)	− 0.30	0.006**
FV875 (775–875 μm^2^)	− 0.14	0.227
FV1000 (900–1000 μm^2^)	− 0.07	0.531
FV1125 (1025–1125 μm^2^)	0.004	0.973

*FV* choriocapillaris flow void.

^†^Spearman's rank correlation coefficient. **P < 0.01.

In a comparison among the CVH[+], CVH[−] and control groups, the number of FVs was significantly lower in the CVH[+] group compared to the control group at the following intervals: FVall (P = 0.004), FV500 (P = 0.001), FV625 (P = 0.001) and FV750 (P = 0.020). No difference was observed among the groups in FV875 (P = 0.217), FV1000 (P = 0.513) or FV1125 (P = 0.522). There were no differences between the CVH[+] and CVH[−] groups, or between CVH[−] and control group at any interval (all P > 0.05) (Table 4).

**Table 4:** The number of the flow void in different CVH status.

Intervals of FV sizes	Control (n = 31) (median, QD)	CVH[−] (n = 15) (median, QD)	CVH[+] (n = 35) (median, QD)	P value Control vs CVH[−]	P value CVH[−] vs CVH[+]	P value Control vs CVH[+]
FVall (0–25,000 μm^2^)	9297.0, 2063.0	8525.0, 1968.0	8240.0, 1043.0	P = 1.000	P = 1.000	P = 0.004**
FV500 (400–500 μm^2^)	4153.0, 1455.0	3015.0, 1240.0	2851.0, 274.0	P = 0.083	P = 1.000	P = 0.001**
FV625 (525–625 μm^2^)	1704.0, 335.0	1518.0, 265.0	1442.0, 153.0	P = 0.347	P = 1.000	P = 0.001**
FV750 (650–750 μm^2^)	1170.0, 224.0	1064.0, 250.0	1050.0, 155.0	P = 0.347	P = 1.000	P = 0.020*
FV875 (775–875 μm^2^)	768.0, 134.0	793.0, 97.0	769.0, 116.0	P = 0.217	P = 0 .217	P = 0.217
FV1000 (900–1000 μm^2^)	551.0, 119.0	563.0, 86.0	537.0, 109.0	P = 0.513	P = 0.513	P = 0.513
FV1125 (1025–1125 μm^2^)	372.0, 126.0	391.0, 60.0	369.0, 110.0	P = 0.522	P = 0.522	P = 0.522

*QD* quartile deviation, *FV* choriocapillaris flow void, *Control* age-matched control group, *CVH[−]* PCV fellow eyes without choroidal vascular hyperpermeability, *CVH[+] P*CV fellow eyes with choroidal vascular hyperpermeability.

Independent-Samples Median Test, adjusted by the Bonferroni correction. *P < 0.05, **P < 0.01.

### The ROC curve of the CVH[+] and control groups

**Figure 5:**
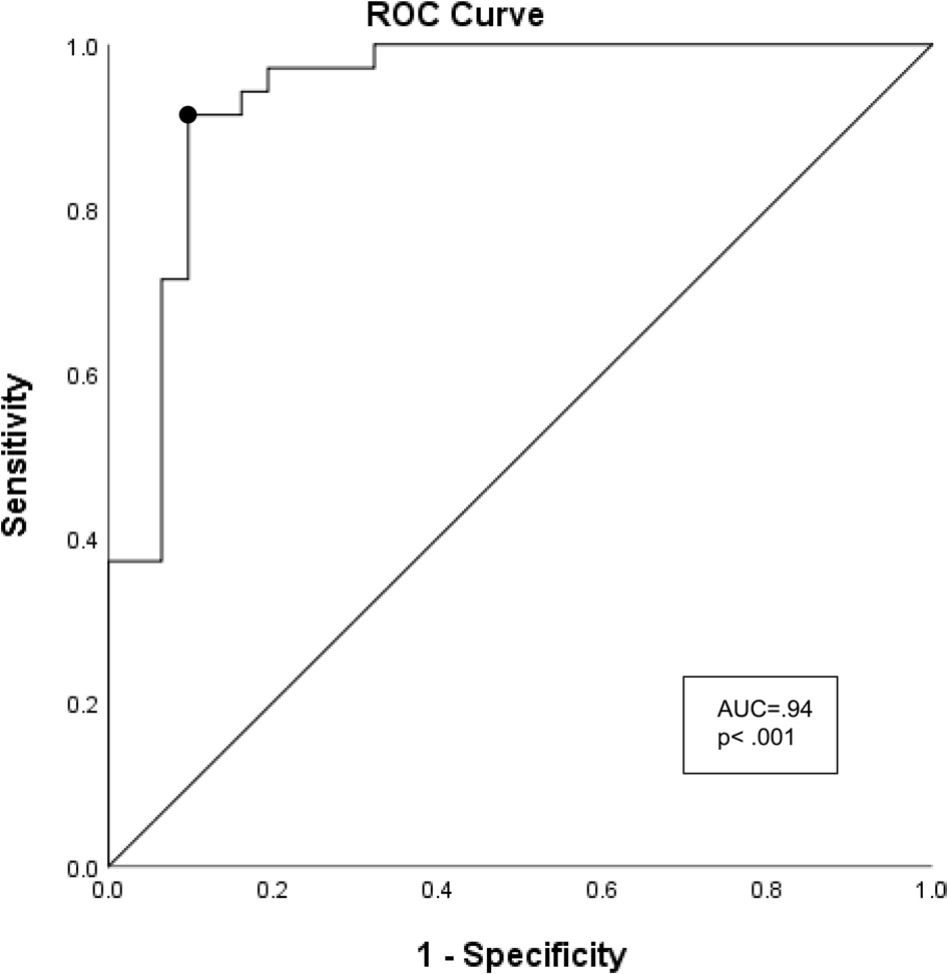
The receiver operating characteristic curve of the fellow eyes of PCV with choroidal vascular hyperpermeability and controls. The circled point is the best cut-off point. Youden index = 0.56, sensitivity = 91.4%, specificity = 86.0%. The area under the curve (AUC) = 0.94 (95% CI 0.88–1.00) (P < 0.001).

Based on the Youden index, the cut-off points of the FVs for predicting fellow eyes in PCV patients with CVH was 0.56, with a sensitivity of 91.4%, specificity of 86.0%, and an area under the curve (AUC) of 0.94 (95% CI 0.88–1.00) (P < 0.001) (Fig. 5).

## Discussion

In our retrospective study, the number of FVs smaller than 750 μm^2^ substantially decreased in the PCVF group compared to the control group; whereas, the total FV area did not differ between the two groups. Most of the PCVF showed CVH (70%; 35 of 50). The status of CVH negatively correlated to the number of FVs.

This is the first study to determine the difference between the fellow eyes of PCV and control eyes using a non-invasive, commercially available SS-OCTA instrument.

Previous studies of ICGA have shown that the pathological changes of PCV and central serous chorioretinopathy have a similar choroidal vascular circulatory disturbance^16^. For the very early stage of the PCV which the choroid shows no pathological changes, recent researches^4,5,17^ cannot tell the difference. However, we found that FV had decreased in the fellow eyes of PCV compared to the controls.

There were different protocols of CC slab obtained by OCTA^12,18–21^. We decided on a protocol that has been implemented in other studies as the standard of our research^12^. We then used our previously published FV analysis method which involved grouping the FV by size and performing statistical analyses on each group^11^. Our grouping analysis strategy can effectively detect the difference of FV in specific intervals which was not identified in previous studies.

**Figure 6:**
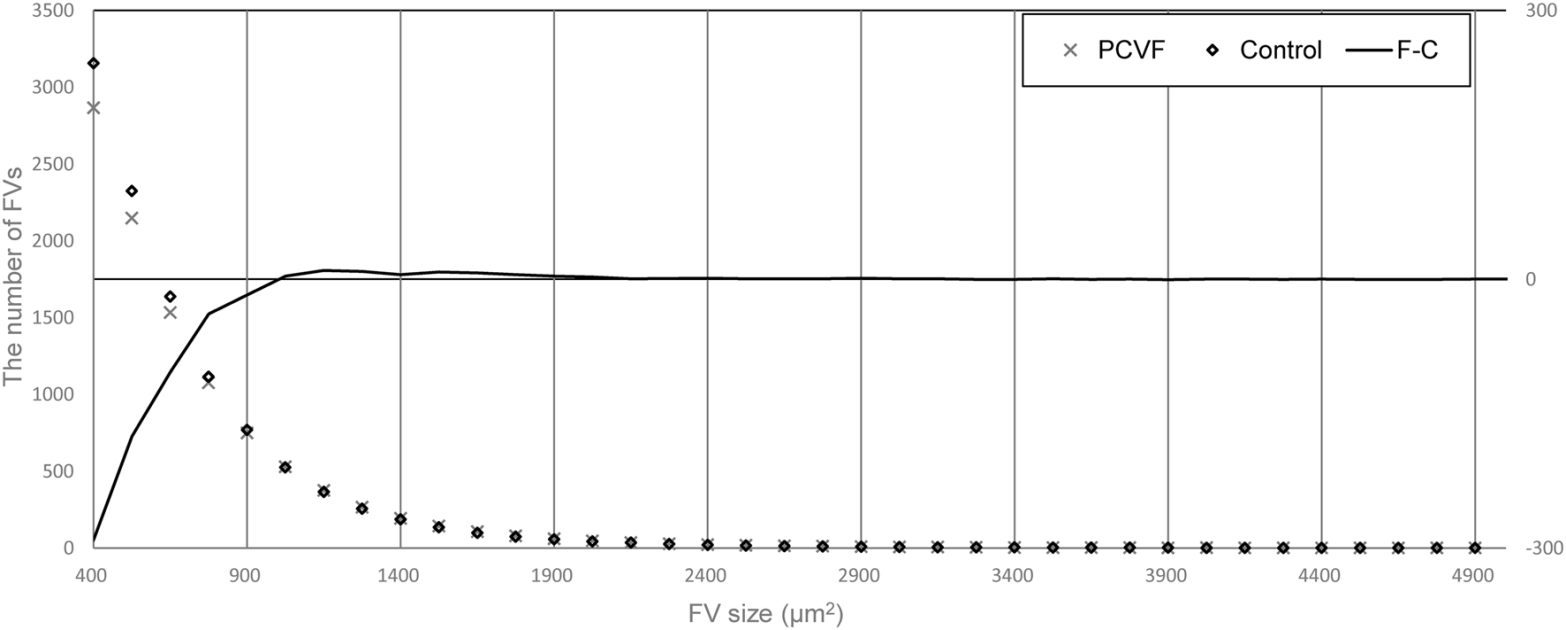
Demonstration of the FV distribution in a fellow eye of PCV with CVH and a control eye. The plot shows the typical power-law distribution of FV. The small sizes had relatively large numbers. The line shows the difference between the two eyes. Our strategy focused on a specific range, which avoids statistical biases due to the large disparity in numbers. *PCVF* PCV fellow eyes, *Control* age-matched control group, *F–C* difference between the PCVF and control groups.

Due to the power-law distribution (Fig. 6), there was a relatively larger number of smaller-sized FV, whereas larger FVs were of a relatively smaller number^22^. If a pathological change occurs in the smaller-sized FV, average-sized FVs may not be affected; on the other hand, if a pathological change occurs in the larger FVs, it was too scarce to affect the total number of FVs. Evaluating the number of grouped FV sizes has another advantage, in that drusen are relatively large in size and frequently appear in PCV^23^. The drusen area can decrease the penetration of OCTA laser to underestimating the FV density^20^. Our grouping strategy can reduce the influence of drusen by calculating the number of FVs rather than the FV area.

The upper limit of the interval in our study was 1125 μm^2^. Due to the power-law distribution^24^, an FV larger than 1125 μm^2^ contained less than 5% of the total, which were excluded from the statistics. An FV smaller than 750 μm^2^ in PCVF was lower in number compared to control group because a small FV is more sensitive to radius changes. For example, when the radius is reduced by 1 μm in two FVs with original areas of 20 μm^2^ and 1125 μm^2^, the areas are reduced by 40% and 6%, respectively. Therefore, FVs of all sizes will be affected when hyperpermeability occurs; however smaller FVs are more substantially affected.

The decreasing size of the FV represents an increase in the blood flow signal area, which might be due to the hyperpermeability of CC, an increase of vessel diameter, or an increase in blood flow.

Recently, OCTA has been effectively detect typical pathological manifestations of PCV^25^. Luo et al. reported similar results to ours in that the proportion of the choriocapillaris flow deficit area of PCV fellow eyes has a decreasing trend, but there was no statistical difference compared with an age-matched control group^5^. However, in the present research, the number of FVs in small sizes was lower compared to that of the controls, while the total area had no difference.

We believe that the insignificant difference in FV in Luo’s research was due to the proportion area analysis strategy or the impact of underlying diseases, which may have resulted in an overall decrease in trend but was not statistically significant. Choroidal circulation can change due to many systemic and ocular factors, such as age^26–28^, choroidal thickness^29^, intraocular pressure^30,31^, and systemic diseases^32–36^. These factors may also change the morphology of the CC, resulting in FV changes. The previous research also revealed that the size of FV in diabetic retinopathy eyes is larger than that in control elderly eyes, and this trend positively correlated with the severity of diabetic retinopathy^32–34^. Hypertension can also increase the size and number of FVs^35–37^. Eyes with advanced Age-Related Eye Disease Study Category stage (AREDS stage) will cause an increase in the size and number of FVs^38,39^. We excluded subjects who had suspected drusen or RPE abnormality on the B-scan, or diseases that had an impact on the CC structure. It can be considered that the different number of FV was not due to from age or any other systematic diseases.

Previous histopathological studies have reported that the CC of the eyes with advanced PCV can have plumped endothelial cells, primitive or narrow lumen, or dilated vessels^40–42^. Our results showed that the reduction of the CVH status negatively correlated with number of FVs. However, the number of FVs of CVH[−] was between the number of those of CVH[+] and the control group, and showed no significant difference (Table 4). There are three possibilities. (1) those in the CVH[−] group are at an earlier stage of PCV than those in the CVH[+] group, and the severity of CVH does not reach an observable level. (2) Although most of the fellow eyes of PCV tend to develop into PCV in the future, not all do. The CVH[−] are normal eyes at the beginning and will not develop into PCV. (3) The number of CVH[−] eyes was too low to achieve significance.

**Figure 7:**
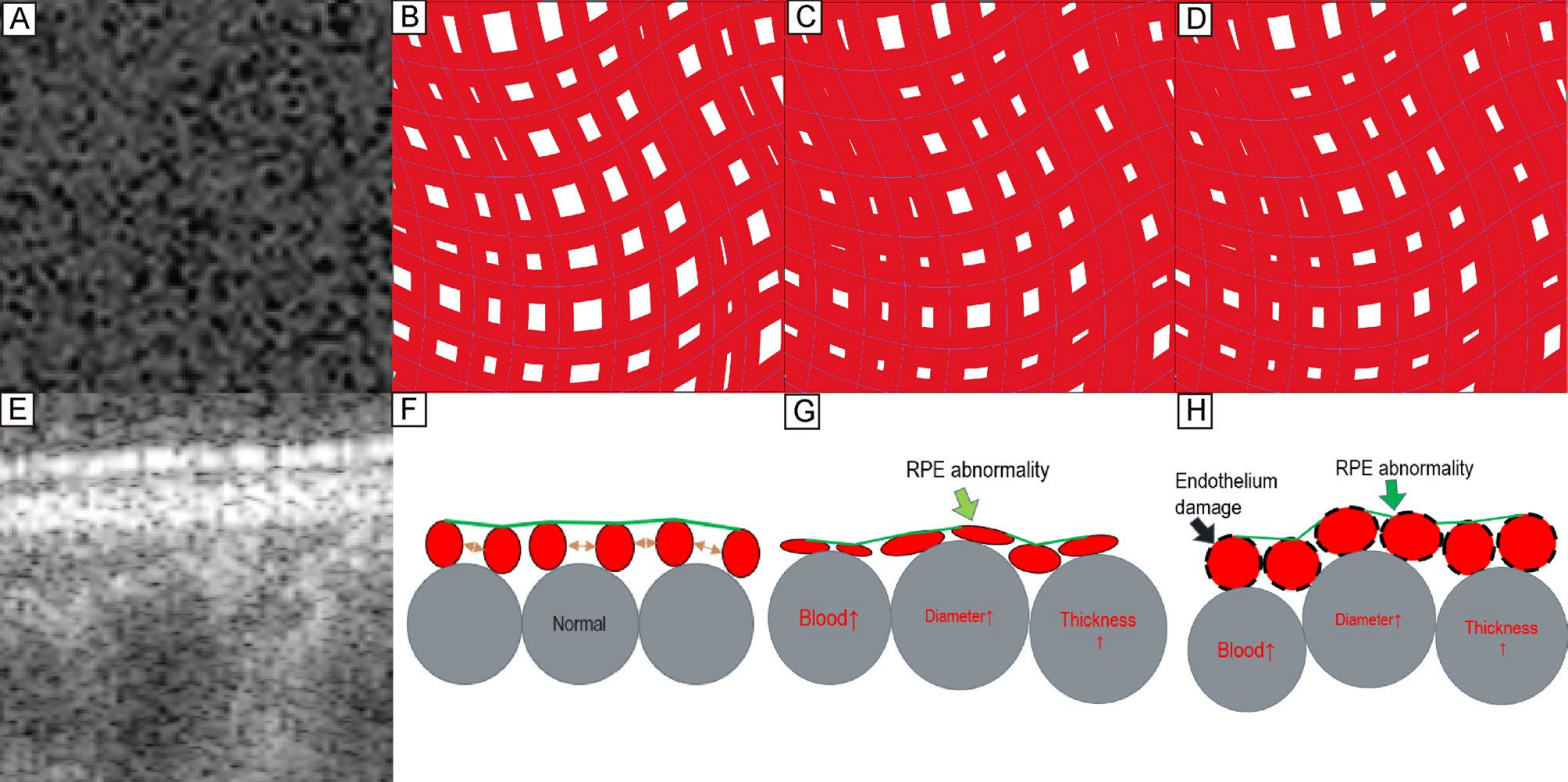
The demonstration of the how the lumen shape of choriocapillaris changes affect the FV on OCTA. (**A**,**E**) OCT angiography image and corresponding B-scan image centered at the fovea. (**B**–**D**) sketches simulating the CC slab with different lumen shapes. The blue line represents the center of each CC vessel. (**B**–**D**) contain the same number of vessels. The red area represents blood flow, which is the light-colored area in (**A**). The white area represents FV, which is the dark area in (**A**). (**F**–**H**) sketches simulating the B-scan with different lumen shapes. Regardless of whether CC is squeezed or dilated on B-scan, similar results will be seen on the OCTA slab and lead to similar FV results. *CC* choriocapillaris, *FV* choriocapillaris flow void.

CVH is more commonly seen in eyes with pachychoroid^43^, but it also occurred in few PCV and fellow eyes^44–49^. A previous study of CVH using monkeys and rabbits had proved that the dye of ICGA was exuded and the CVH was formed due to vascular endothelial damage^50^, suggesting that CVH exhibits vessel abnormalities at the innermost choroid. Based on the mechanism of OCTA, the blood flow signals came from the movement of red blood cells^6^, and the decrease of the area without blood flow signals (FV) means that the area occupied by red blood cells increased. However, red blood cells should not exist outside the vessels. In other words, the diameter of the CC that parallel to the RPE was increased (Fig. 7).

One possibility is that due to the vasodilation of the choroidal vessels, the CC was squeezed toward the RPE, causing mechanical deformation of the CC (the diameter of the CC perpendicular to the RPE decreased and the diameter parallel to the RPE increased). As the CC lumen is squeezed, local blood flow can be slowed down and occurred turbulence. Slow blood flow and turbulence cause thrombosis and may be further related to the pathogenesis of PCV (Fig. 7G). Another possibility is that the diameters perpendicular to the RPE and parallel to the RPE were both dilated, induced CC endothelial damage, which causes the leakage of ICGA dye, and exhibited CC hyperpermeability. At the same time, due to endothelial damage, the concentration of vascular endothelial growth factors increased. Which in turn further promoted vasodilation and eventually formed pachychoroid disease (Fig. 7H).

These two assumptions have the same characteristics on the CC slab of SS-OCTA: the diameter parallel to the CC slab increased, and the FV became smaller (Fig. 7C,D).

Our result shows that the hyperpermeability of PCVF occurs earlier than detectable RPE abnormalities on B-scans of SS-OCT.

Based on the Youden index, our model has high sensitivity (91.4%) and specificity (86.0%). There is currently no method to detect specific signs before the first eye of PCV onset. Our model shows a reliable prediction (AUC = 0.94) of PCV using non-invasive, dye-free and commercially available OCTA instruments. Therefore, this model, with the number of FVs as a predictor, is helpful. The change of FVs can give a clue for the early screening of PCV in the future.

The limitations of present study are its retrospective nature and small sample size. Although we strictly controlled the underlying conditions of both groups, fellow eyes of RVO may have had some unknown conditions that could have limited the generalizability of our findings in clinical practice. We used the watershed method to separate the large FVs, which may lead to underestimation of the number of large FVs and overestimation of the number of the small FVs. Moreover, CVH can also occur in other diseases, including central serous chorioretinopathy. Further research is necessary to specifically distinguish PCV with CVH from other diseases.

In conclusion, the number of FV was significantly lower in fellow eyes of PCV than in eyes with normal ocular blood circulation. In most of the PCV fellow eyes of this research, CVH occurred, which should be the earliest detectable sign of PCV when investigations yield normal results..

## Data Availability

The datasets generated and analyzed during the current study are available from the corresponding author on reasonable request.

## References

[1] Yannuzzi LA, Sorenson J, Spaide RF, Lipson B (1990). Idiopathic polypoidal choroidal vasculopathy (IPCV). Retina.

[2] Spaide RF, Yannuzzi LA, Slakter JS, Sorenson J, Orlach DA (1995). Indocyanine green videoangiography of idiopathic polypoidal choroidal vasculopathy. Retina.

[3] Sho K (2003). Polypoidal choroidal vasculopathy: Incidence, demographic features, and clinical characteristics. Arch. Ophthalmol..

[4] Spaide RF, Ledesma-Gil G (2020). Choriocapillaris vascular parameters in normal eyes and those with pachychoroid with and without disease. Retina.

[5] Luo M (2020). Comparison of choriocapillary flow density between fellow eyes of polypoidal choroidal vasculopathy and neovascular age-related macular degeneration. BMC Ophthalmol..

[6] Kashani AH (2017). Optical coherence tomography angiography: A comprehensive review of current methods and clinical applications. Prog. Retin Eye Res..

[7] Olver JM (1990). Functional anatomy of the choroidal circulation: Methyl methacrylate casting of human choroid. Eye (Lond.).

[8] Yoneya S, Tso MO (1987). Angioarchitecture of the human choroid. Arch. Ophthalmol..

[9] Fryczkowski AW (1994). Anatomical and functional choroidal lobuli. Int. Ophthalmol..

[10] Schindelin J (2012). Fiji: An open-source platform for biological-image analysis. Nat. Methods.

[11] Huajui Wu (2021). A modified measuring method to investigate the choriocapillaris flow void of polypoidal choroidal vasculopathy with swept source optical coherence tomography angiography. Quant. Imaging Med. Surg..

[12] Spaide RF, Fujimoto JG, Waheed NK (2015). Image artifacts in Optical coherence tomography angiography. Retina.

[13] Zhang A, Zhang Q, Wang RK (2015). Minimizing projection artifacts for accurate presentation of choroidal neovascularization in OCT micro-angiography. Biomed. Opt. Express.

[14] Neerad, P., Sumit, M., Ashish, S. & Madhuri, J. Adaptive local thresholding for detection of nuclei in diversity stained cytology images. In *2011 International Conference on Communications and Signal Processing* 218–220 (2011).

[15] Soille P, Vincent L (1990). Determining Watersheds in Digital Pictures Via Flooding Simulations.

[16] Guyer DR (1994). Digital indocyanine-green videoangiography of occult choroidal neovascularization. Ophthalmology.

[17] Baek J, Cheung CMG, Jeon S, Lee JH, Lee WK (2019). Polypoidal Choroidal vasculopathy: Outer retinal and choroidal changes and neovascularization development in the fellow eye. Invest. Ophthalmol. Vis. Sci..

[18] Chu Z, Gregori G, Rosenfeld PJ, Wang RK (2019). Quantification of choriocapillaris with optical coherence tomography angiography: A comparison study. Am. J. Ophthalmol..

[19] Byon I, Nassisi M, Borrelli E, Sadda SR (2019). Impact of slab selection on quantification of choriocapillaris flow deficits by optical coherence tomography angiography. Am. J. Ophthalmol..

[20] Chu Z (2020). Quantification of choriocapillaris with phansalkar local thresholding: Pitfalls to avoid. Am. J. Ophthalmol..

[21] Chu Z, Zhang Q, Gregori G, Rosenfeld PJ, Wang RK (2020). Guidelines for Imaging the choriocapillaris using OCT angiography. Am. J. Ophthalmol..

[22] Newman MEJ (2005). Power laws, Pareto distributions and Zipf's law. Contemp. Phys..

[23] Spaide RF (2018). Disease expression in nonexudative age-related macular degeneration varies with choroidal thickness. Retina.

[24] Spaide RF (2016). Choriocapillaris flow features follow a power law distribution: Implications for characterization and mechanisms of disease progression. Am. J. Ophthalmol..

[25] Kim K (2020). A comparison study of polypoidal choroidal vasculopathy imaged with indocyanine green angiography and swept source OCT angiography. Am. J. Ophthalmol..

[26] Yun C, Nam KT, Park S, Hwang SY, Oh J (2020). Features of the choriocapillaris on four different optical coherence tomography angiography devices. Int. Ophthalmol..

[27] Zheng F (2019). Age-dependent changes in the macular choriocapillaris of normal eyes imaged with swept-source optical coherence tomography angiography. Am. J. Ophthalmol..

[28] Sacconi R (2019). Quantitative changes in the ageing choriocapillaris as measured by swept source optical coherence tomography angiography. Br. J. Ophthalmol..

[29] Rochepeau C (2018). Optical coherence tomography angiography quantitative assessment of choriocapillaris blood flow in central serous chorioretinopathy. Am. J. Ophthalmol..

[30] Mammo Z (2016). Quantitative optical coherence tomography angiography of radial peripapillary capillaries in glaucoma, glaucoma suspect, and normal eyes. Am. J. Ophthalmol..

[31] Yarmohammadi A (2016). Optical coherence tomography angiography vessel density in healthy, glaucoma suspect, and glaucoma eyes. Invest. Ophthalmol. Vis. Sci..

[32] Thompson IA, Durrani AK, Patel S (2019). Optical coherence tomography angiography characteristics in diabetic patients without clinical diabetic retinopathy. Eye (Lond.).

[33] Cao D (2018). Optical coherence tomography angiography discerns preclinical diabetic retinopathy in eyes of patients with type 2 diabetes without clinical diabetic retinopathy. Acta Diabetol..

[34] Dodo Y (2017). Clinical relevance of reduced decorrelation signals in the diabetic inner choroid on optical coherence tomography angiography. Sci. Rep..

[35] Bosch AJ (2017). Retinal capillary rarefaction in patients with untreated mild-moderate hypertension. BMC Cardiovasc. Disord..

[36] Sun C (2020). Systemic hypertension associated retinal microvascular changes can be detected with optical coherence tomography angiography. Sci. Rep..

[37] Jumar A (2016). Improvement in retinal capillary rarefaction after valsartan treatment in hypertensive patients. J. Clin. Hypertens. (Greenwich).

[38] Mullins RF, Johnson MN, Faidley EA, Skeie JM, Huang J (2011). Choriocapillaris vascular dropout related to density of drusen in human eyes with early age-related macular degeneration. Invest. Ophthalmol. Vis. Sci..

[39] Biesemeier A, Taubitz T, Julien S, Yoeruek E, Schraermeyer U (2014). Choriocapillaris breakdown precedes retinal degeneration in age-related macular degeneration. Neurobiol. Aging.

[40] Okubo A, Sameshima M, Uemura A, Kanda S, Ohba N (2002). Clinicopathological correlation of polypoidal choroidal vasculopathy revealed by ultrastructural study. Br. J. Ophthalmol..

[41] Lafaut BA, Aisenbrey S, Van den Broecke C, Bartz-Schmidt KU, Heimann K (2000). Polypoidal choroidal vasculopathy pattern in age-related macular degeneration: A clinicopathologic correlation. Retina.

[42] Moussa K (2017). Polypoidal choroidal vasculopathy: A clinicopathologic study. Retin. Cases Brief Rep..

[43] Borooah S (2020). Pachychoroid spectrum disease. Acta Ophthalmol..

[44] Yanagi Y (2018). Choroidal vascular hyperpermeability as a predictor of treatment response for polypoidal choroidal vasculopathy. Retina.

[45] Lee J, Byeon SH (2019). Prevalence and clinical characteristics of pachydrusen in polypoidal choroidal vasculopathy: Multimodal image study. Retina.

[46] Koizumi H, Yamagishi T, Yamazaki T, Kinoshita S (2013). Relationship between clinical characteristics of polypoidal choroidal vasculopathy and choroidal vascular hyperpermeability. Am. J. Ophthalmol..

[47] Chung SE, Kang SW, Kim JH, Kim YT, Park DY (2013). Engorgement of vortex vein and polypoidal choroidal vasculopathy. Retina.

[48] Ryu G, Moon C, van Hemert J, Sagong M (2020). Quantitative analysis of choroidal vasculature in polypoidal choroidal vasculopathy using ultra-widefield indocyanine green angiography. Sci. Rep..

[49] Sasahara M (2006). Polypoidal choroidal vasculopathy with choroidal vascular hyperpermeability. Am. J. Ophthalmol..

[50] Yoshioka H (1991). The etiology of central serous chorioretinopathy. Nippon Ganka Gakkai Zasshi.

